# Daily changes in the electric behavior of weakly electric fish naturally persist in constant darkness and are socially synchronized

**DOI:** 10.1242/bio.036319

**Published:** 2018-10-19

**Authors:** Adriana Migliaro, Victoria Moreno, Paul Marchal, Ana Silva

**Affiliations:** 1 Laboratorio de Neurociencias, Facultad de Ciencias, Universidad de la República, Montevideo 11400, Uruguay; 2 Unidad Bases Neurales de la Conducta, Instituto de Investigaciones Biológicas Clemente Estable, Ministerio de Educación y Cultura, Montevideo 11600, Uruguay; 3 Ecole Normale Superieure de Lyon, Université Claude Bernard, Lyon 69007, France

**Keywords:** Daily rhythms, Electric behavior, Electric fish, Temperature, Environmental influences, Social context

## Abstract

Daily rhythms allow anticipation of changes and allocation of energy to better cope with predictable events. Rhythms in behavior result from a complex combination of physiological processes timed by the nervous system and synchronized with external information. We aimed to understand how rhythmic behaviors arise in nature, when weakly electric fish are exposed to cyclic environmental influences and social context. *Gymnotus omarorum* is a South American nocturnal pulse-type gymnotiform. Its electric behavior encodes information about species, sex and physiological state. The rate of emission of the electric organ discharge (EOD-BR) is modulated by exploratory activity and by physical and social environmental stimuli. We show that the EOD-BR increases during the night in the natural habitat even in individuals maintained in constant dark conditions. Locomotor activity is higher at night, however the nocturnal increase of EOD-BR still occurs in motionless fish, demonstrating an independent origin for the locomotor and electric components of exploratory behavior. When fish are observed in nature, social context exerts a synchronizing role on electric behavior. *G. omarorum* emerges as an exciting wild model for the study of daily rhythms arising in the complexity of the real world, integrating environmental, physical and social cues in the modulation of rhythmic behavior.

## Abstract (precis)

**Summary:** The nocturnal increase of electric behavior in *Gymnotus omarorum* is analyzed in the wild, in constant darkness and social isolation. This daily trait is independent of locomotor activity and modulated by social context.

## INTRODUCTION

Circadian rhythms allow living organisms to anticipate changes and allocate energy to cope with predictable events. Current knowledge about circadian systems provides deep insight into temporal dynamics as well as in molecular processes subserving rhythmicity. However, these analyses have mostly been carried out in precisely controlled laboratory conditions, which lack the complexity of natural environmental constraints, leading to interpretations that fail to include the whole range of modulating factors operating in nature (Tomotani et al., 2012). In our quest to understand the adaptive role of circadian systems we should aim to broaden our scope by including novel animal models and rhythmic variables that arise in natural environments, synchronous with natural environmental cues. Current chronobiological research thus faces a double challenge: on the one hand, to be considered a proper circadian rhythm, daily variations of a given trait should fulfil a number of requirements that include persistence in free-running conditions of illumination as well as independence from social and environmental changes. On the other hand, the analysis of how biological clocks adapt to constantly changing environments ­– i.e. in the real world – needs to be added to the agenda of chronobiological studies (Kronfeld-Schor et al., 2013).

Nocturnal fishes of the South American order Gymnotiformes are characterized by the emission of species-specific weak electric discharges that serve electrosensory and electrocommunicative purposes (Lissmann, 1958; Stoddard, 2002). These fish exhibit a typical electric behavior consisting of pulse discharges (the ‘electric organ discharge’; EOD) emitted continuously by a specialized electric organ. The medullary pacemaker nucleus commands the basal rate of the EOD (EOD-BR) (Bennett et al., 1967) and receives modulatory central connections from prepacemaker structures. Rate modulations of the EOD depend on several environmental factors, in particular and very importantly, on water temperature, in a direct relationship that is sustained by the influence of temperature on physiological processes (Ardanaz et al., 2001). The EOD is a behavioral display that encodes information (in waveform and frequency domains) about an individual's species identity, sex and physiological state (Bullock et al., 2005). In addition, the EOD is the physical carrier of perceptually relevant sensory information (Aguilera et al., 2001). Arousal states in weakly electric fish imply an increase in EOD basal rate, providing a greater amount of available sensory information. Exploratory movements, novelty detection, escape responses and even volition are associated with increases in EOD-BR. Arousal is expected to coincide with the activity phase and thus EOD-BR is expected to be higher during the night (Black-Cleworth, 1970; Jun et al., 2014; Migliaro and Silva, 2016; Silva et al., 2007). Two main questions are then to be addressed: (1) is this nocturnal increase in EOD-BR also observed in nature? (2) Is the nocturnal increase in EOD-BR an independent trait or only a consequence of fish locomotion?

*Gymnotus omarorum* is a pulse type gymnotiform widely distributed in Uruguay (Richer-de-Forges et al., 2009; Silva et al., 2003) and an excellent model system for the study of modulation of the biological clock by environmental influences. It is a seasonal breeder that displays intra and inter*-*sexual aggression all year round (Batista et al., 2012; Silva et al., 2003), it exhibits high-site fidelity and shows neighbor intolerance consistent with territoriality (L. Zubizarreta, Facultad de Medicina, Universidad de la República, Montevideo, personal communication). Isolated individuals in laboratory settings show a melatonin-dependent nocturnal increase in EOD-BR during the first hour of darkness under a 12:12 light-dark cycle and constant temperature (Migliaro and Silva, 2016).

Our aim was to analyze if the nocturnal increase in EOD-BR, previously recorded in laboratory settings in *G. omarorum*, is a trait that can also be observed in nature. To do so, we first confirmed that though these nocturnal animals increase their exploratory activity during nighttime, the nocturnal increase in EOD-BR persists even in sheltered fish. Secondly, we found a marked daily rhythm in EOD-BR in freely moving fish recorded in their natural habitat, which is independent of light-dark cues as it occurs in constant darkness.

## RESULTS

### Nocturnality in *G. omarorum*

**Fig. 1.**
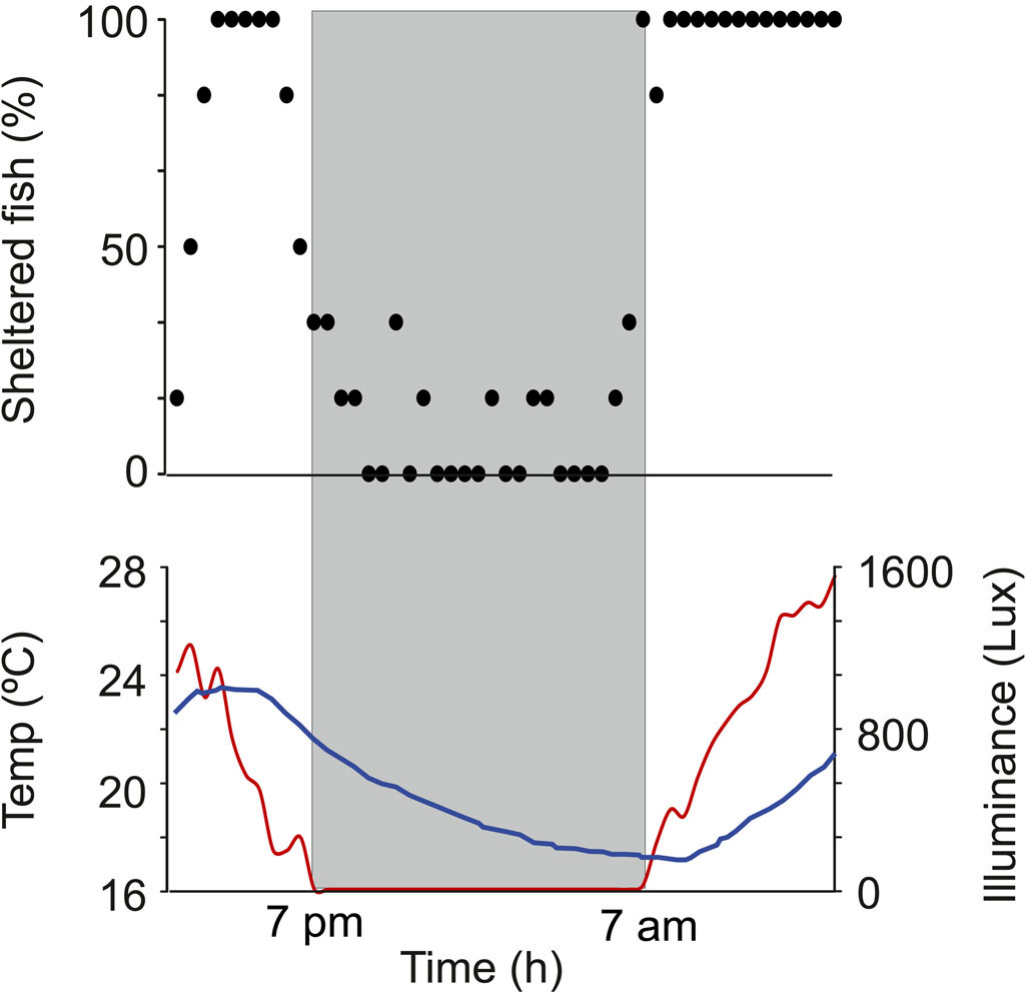
**Daily changes in sheltering behavior.** (Upper panel) Percentage of sheltered fish through a 24 h period. Each black dot accounts for the percentage of isolated fish (*n*=6) found in one of the 12 available shelters each 30 min. (Lower panel) Red line shows mean light intensity under the water. Blue line shows mean water temperature. Mean values from the six consecutive days of recording. Gray shadow signals the dark period (19:00 h–07:00 h).

**Fig. 2.**
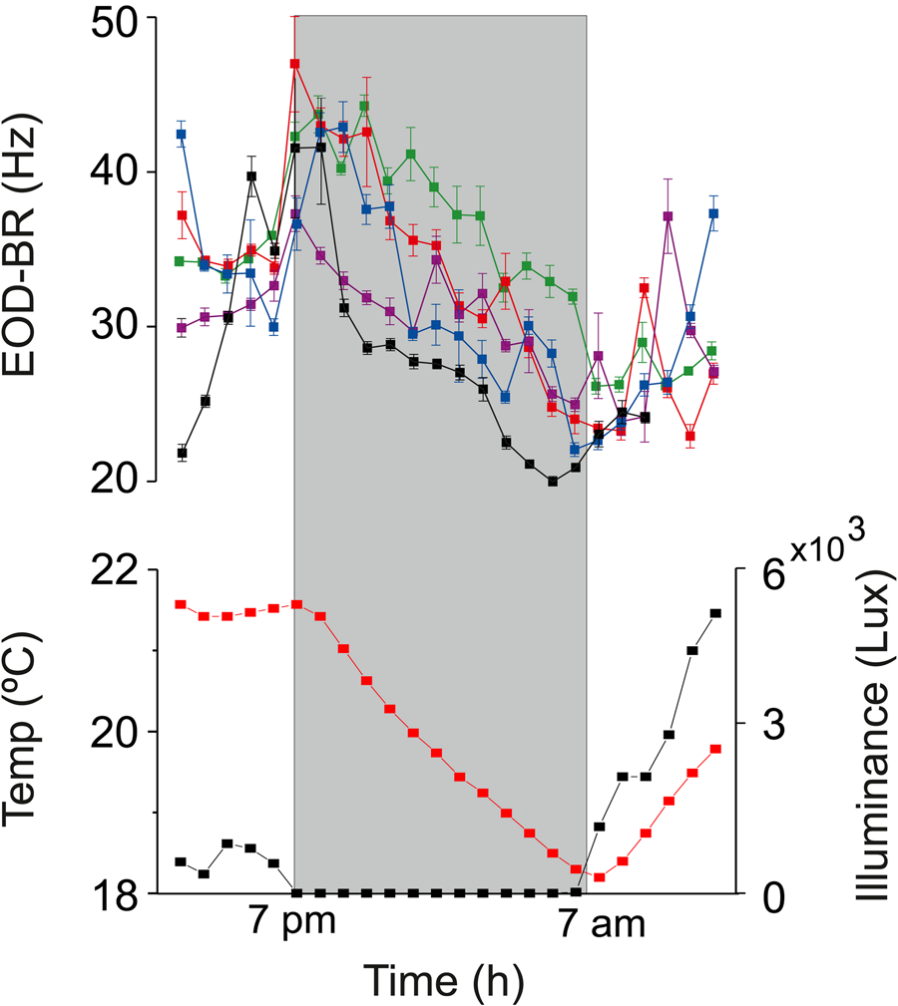
**24 h recordings of EOD-BR of isolated animals (*n*=5) in a semi-natural setup.** (Upper panel) Fish were recorded each hour only when motionless (EOD-BR values represent the median for±MAD). Each line in the upper panel shows EOD-BR for a single fish. (Lower panel) Water temperature (black) and light intensity (red). Gray shadow signals the dark period (19:00 h–07:00 h).

*G. omarorum* has long been considered nocturnal based on behavioral analysis that took into account their general increase in activity during the night. Our first aim was to determine if active exploration was enhanced during nighttime as an individual trait. When isolated animals are presented with an enriched environment to explore and exposed to the natural photoperiod, sheltering behavior occurs mainly during daytime. Fig. 1 shows the percentage of fish recorded inside shelters at 30 min intervals during a 24 h period. During the night (19:00 h–07:00 h) 0–30% of fish are sheltered, whereas during the day, 100% of fish are sheltered most of the time (*n*=6; Wilcoxon test, *P*=0.02). This experiment shows that the motor component of exploratory behavior increases during the night. Exploratory activity in this species is also characterized by an increase in EOD-BR; an increase in sampling frequency that enhances perception. In order to break down the complexity of this joint increase in movement and EOD-BR, we recorded daily changes in EOD-BR in sheltered fish in semi-natural settings (Fig. 2). EOD-BR is higher during nighttime; more specifically, at sunset, EOD-BR values are 17.5% higher than in the previous hour [index of BR change (BrIn)=0.175±0.09] (BR_sunset_ versus BR_−60_; *n*=5; Wilcoxon test; *P*=0.04). Since EOD was recorded only if fish were sheltered and thus not swimming, these results show that the nocturnal increase in EOD-BR is independent of the increase in locomotor activity.

### Daily changes of electric behavior in nature

**Fig. 3.**
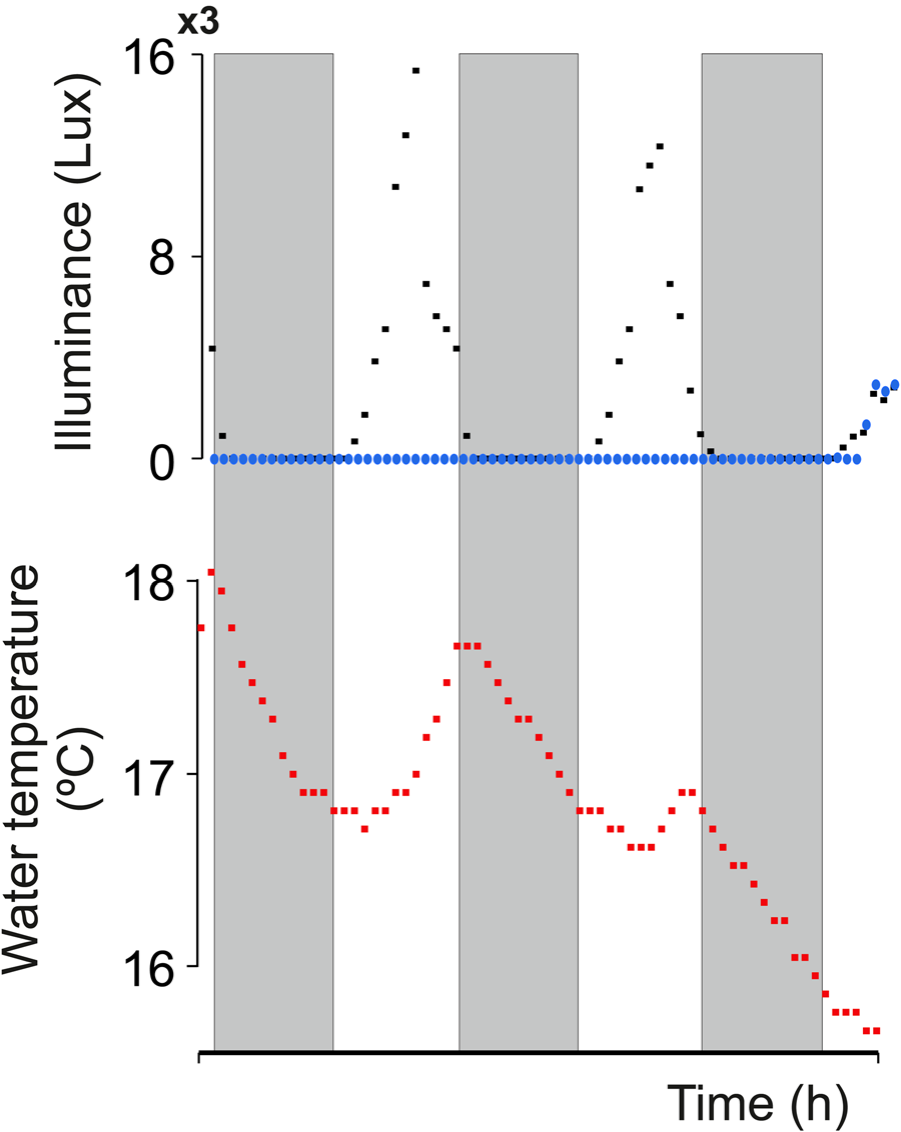
**Light and temperature recordings during 72 h in the natural habitat.** (Upper panel) Light intensity outside (black squares) and inside the water (blue circles). (Lower panel) Water temperature. Gray shadows signal the three dark periods (19:00 h–07:00 h).

**Fig. 4.**
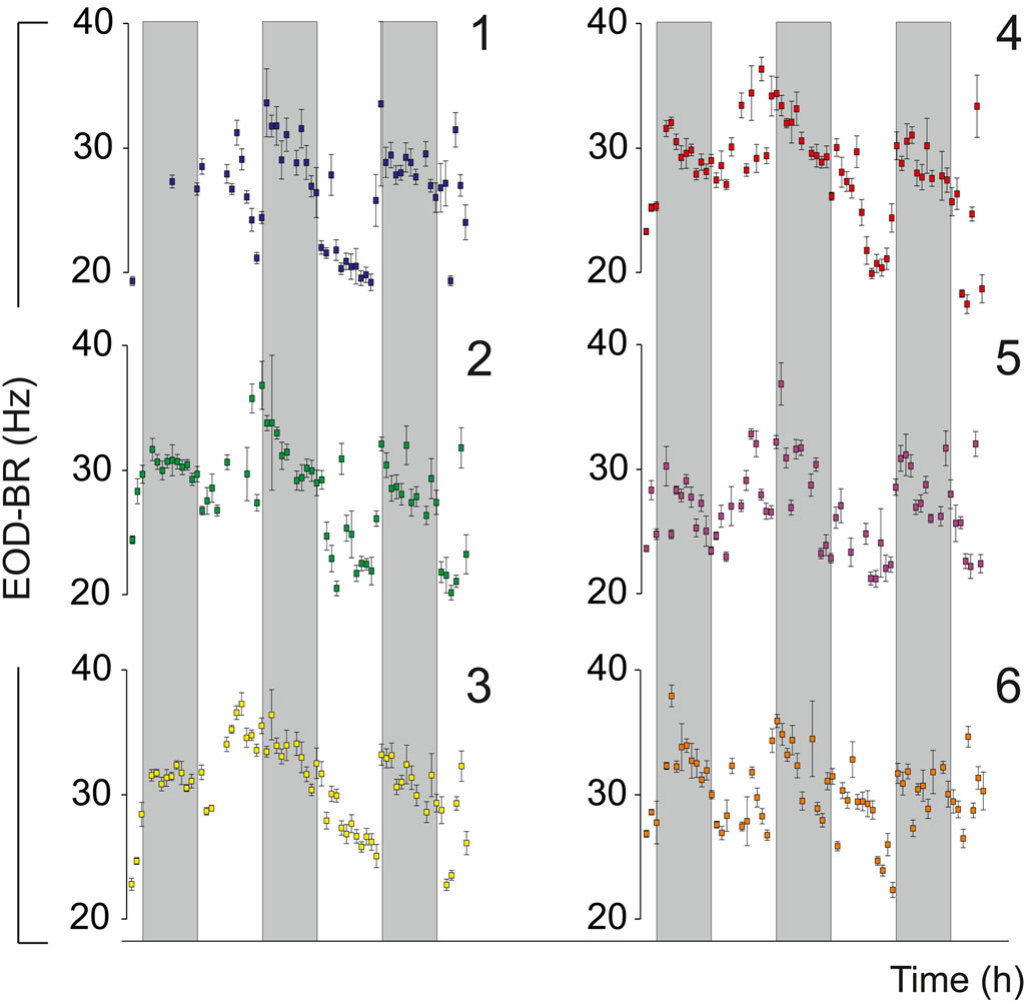
**3 day recordings of EOD-BR in the natural habitat.** The EOD was recorded each hour (EOD-BR values represent the median for±MAD) in six fish (numbered 1–6). Gray shadows signal the three dark periods (19:00 h–07:00 h).

Assessing the daily changes of electric behavior in the wild is mandatory for understanding its regulation in a natural context. Light and temperature changes inside and outside the water were monitored over 72 h in the natural habitat of the fish. While outside light levels followed the obvious day-night rhythm, the environment under the thick vegetation that characterizes electric fish habitats was constantly dark (Fig. 3). Water temperature cycled as expected, rising through the day and peaking towards sunset. Fish were recorded in their natural habitat under dense vegetation with each individual placed in a large plastic mesh. Fish could move within the mesh and were eventually perceived near conspecifics. Fig. 4 shows individual (*n*=6) EOD-BR changes over 72 h. Electric behavior has a clear daily rhythm, rising towards sunset and decaying towards dawn. Mean EOD-BR is 12% basal rate at sunset (BR_sunset_) versus basal rate 60 minutes before sunset (BR_−60_) (BrIn=0.12±0.05) (BR_sunset_ versus BR_−60_; *n*=6; Wilcoxon test, *P*=0.02). Daily rhythmicity was confirmed by cosinor analysis for a 24 h period. Table 1 presents the parameters obtained for each tested fish. It is interesting to note that five out of six recorded fish had maximum basal rate values during the night (indivudual acrophase at night). It is also noteworthy that the one fish in which the cosinor fit failed to be significant was also the fish in which we missed several EOD samples (fish 1 in Fig. 3 and Table 1).

**Table 1.**
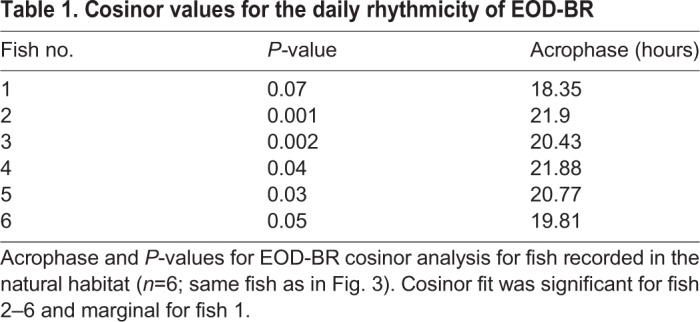
**Cosinor values for the daily rhythmicity of EOD-BR**

### Environmental influences on the nocturnal increase in electric behavior

**Fig. 5.**
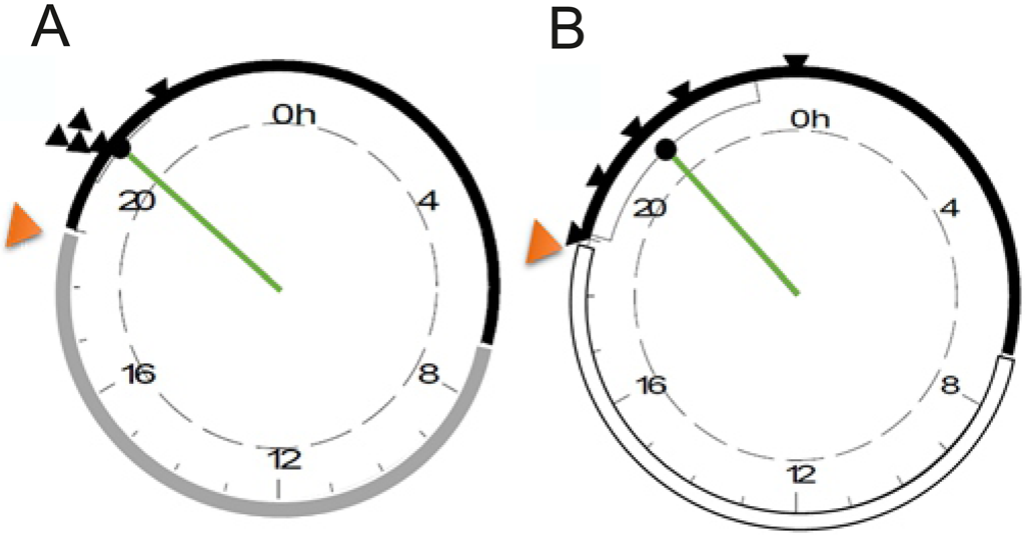
**Daily rhythms in populations recorded in natural and semi-natural settings.** Rayleigh test for fish recorded (A) in the natural (*n*=5), and (B) the semi-natural conditions (*n*=5). Black arrowheads indicate each individual's acrophase, orange arrowheads signal the time of maximum water temperature. Green line shows mean population acrophase and its length represents the Rayleigh radius (r). Dashed circle represents *P*=0.05 confidence level. Photoperiod is depicted by the outline of each circle: black, night; white, day; gray, constant darkness during daytime.

EOD-BR recordings presented here were carried out in two different conditions regarding environmental daily cues. Fish kept in plastic meshes inside the lagoon under natural vegetation (natural condition), were in a constant dark environment. Isolated fish, on the other hand, were kept in plastic containers exposed to daily photoperiodic changes (semi-natural condition), albeit partially submerged in the lagoon and partially covered by vegetation. Possible effects of these different environments on EOD-BR rhythmicity were then analyzed. In the natural scenario, EOD-BR increases towards sunset, peaking on the second or third hour of the night. The acrophase for this group was calculated taking into account only those individuals with a cosinor *P*-value<0.05 (*n*=5). Rayleigh tests for each condition are shown in Fig. 5. Median acrophase for fish recorded in nature is 20:41 h±0.09 h, showing a high degree of synchronization among individuals (r=0.988; *P*=0.002). Fish recorded in the semi-natural experiment show a similar daily rhythm with a very similar population acrophase: 21:00 h (r=0.902; *P*=0.009). However, the individual acrophases are more scattered during a 4 h period and hence show less synchronization than fish in natural conditions.

## DISCUSSION

With the aim of contributing to the understanding of the adaptation of biological clocks to natural life, we provide a novel animal model system in which to analyze daily changes in the wild under the natural constraints of physical and social environmental cues. In this study, we show that *G. omarorum* is a clearly nocturnal fish that increases both its exploratory behavior and its electric behavior during the night. We were also able to demonstrate that the nocturnal electrical arousal is independent of locomotion and occurs under natural constant darkness.

Fresh water South American weakly electric fish are well known as nocturnal animals. Nocturnality has been explored in several species of Gymontiformes with behavioral approaches that focused on locomotor activity, electric behavior and electrocommunication. While early experiments showed a nocturnal increase in locomotor activity and exploratory behavior with a natural photoperiod (Black-Cleworth, 1970; Lissmann and Schwassmann, 1965), more recent reports attempted to demonstrate the circadian nature of nocturnal variations in electric behavior. Zupanc (2001) showed that social electric signals occur during the night or the subjective night when animals are in constant darkness. Stoddard et al. (2007) showed that the basal rate of the EOD has a rhythm of nocturnal increase that persisted in constant light and constant darkness only expressed in social context as animals would lose their rhythm in isolation. Strictly speaking, so far it has not been possible to demonstrate the persistence of an endogenous circadian rhythm of electric behavior in isolated individuals in free running conditions. In this study, we show the emergence of this rhythm in nature, its independence from locomotion and its persistence in constant darkness, albeit in the presence of other cyclic variables such as social context and temperature.

Nocturnality is, of course, an expected adaptation for animals that depends on a main sensory modality whose resolution is independent of environmental light changes. So, while nocturnality of weakly electric fish is not a matter for discussion, the dissection between locomotor and electric arousal during the nocturnal active phase has always been difficult to achieve, given their mutual influences. In other words, as locomotion is associated with exploration and, therefore, to an increase in the demands of electrosensory information (i.e. to an increase of EOD rate), no specific attempts have been made to distinguish daily variations of locomotor and electric displays as separate traits. To do that, the first step of this study was to confirm the nocturnality of *G. omarorum*. In an enriched environment (outdoor tank with multiple shelters), individuals of *G. omarorum* tend to rest during daytime and abandon shelters during the night (Fig. 1).

It is generally accepted that electric behavior is modulated by different environmental factors. Particularly, changes in water temperature induce direct proportional changes in EOD-BR in *G. omarorum* as a result of the thermal dependence of the ectotherm’s physiological processes (Ardanaz et al., 2001). Additionally, in this species, the detection of novel stimuli is associated with a fast and transient (<1 s) increase in EOD rate known as novelty response (Caputi et al., 2003), the dynamics of which depend on the stimulus intensity and its sensory modality (Capurro, 1994; Caputi et al., 2003). The activation of the Mauthner cell, a trigger for escape responses, also generates an abrupt and short (5 s) increase in electric behavior in *G. omarorum* (Falconi et al., 1995). Social context is a strong modulator of EOD rate modifying its basal rate in this species (Perrone and Silva, 2018) as well as influencing the generation of transient modulations, the social electric signals (Hagedorn and Heiligenberg, 1985). These social modulations are mediated by the hypothalamic neuropeptide arginine-vasotocin, which directly influences the discharge rate of the medullary pacemaker nucleus (Perrone et al., 2014). Locomotion is also associated with a general increase in electric behavior as has been documented in several gymnotiform species (Black-Cleworth, 1970; Jun et al., 2014; Kawasaki and Heiligenberg, 1989). Moreover, recent findings highlight the occurrence of transient increases in EOD rate immediately before the beginning of movement, a behavioral display that has been pinpointed as an evidence of volition (Jun et al., 2014). Except for water temperature changes, the factors mentioned so far occur with a greater probability during the night. So, we can pose the question of whether the nocturnal increase of EOD-BR is a genuine independent attribute or if it is secondary to a complex combination of all these diverse stimulating effects. In this study, we were able to demonstrate that the nocturnal increase of EOD-BR stands as an independent trait as it persists through the night even in isolated motionless fish (Fig. 2).

We hypothesize that the nocturnal electric arousal; i.e. the nocturnal increase of EOD-BR, emerges from an endogenously-driven circadian rhythm of EOD-BR. In a previous study, we observed that isolated individuals of *G. omarorum* in laboratory settings show a transient melatonin dependent increase in EOD-BR during the first hour of the night, strongly suggesting a circadian control of electric arousal (Migliaro and Silva, 2016). We now add relevant evidence to support the robustness of the nocturnal increase of EOD-BR as: (1) it occurs in freely moving fish in the wild, confirming its adaptive relevance (Fig. 4); (2) it stands as a locomotor-independent trait (Fig. 2); and (3) it persists in constant darkness in the wild (Fig. 3). It is notable to confirm that while fish stay under a thick layer of aquatic plants that prevents light penetration (Fontanarrosa et al., 2010) EOD-BR swings daily regardless of the lack of environmental light information. Somehow, the constant darkness in which these animals normally live provides a natural experiment to demonstrate that daily variations of EOD-BR do not require photoperiodic information.

Even though these fish live in constant darkness, other environmental temporal information must be available in the wild to allow synchronization of natural rhythms. Besides light, daily changes of environmental temperature have been traditionally recognized as reliable entrainers of endogenous circadian rhythms and are particularly important for ectothermic animals (Pittendrigh, 1960). Seasonal water temperature changes have been reported to trigger the onset of the breeding season in a sympatric gymnotiform species (Quintana et al., 2004; Silva et al., 2003). In these reports, manipulation of water temperature induced gonadal maturation, sexual dimorphism and reproductive behavior, thus synchronizing the reproductive cycle. In *G. omarorum*, daily variations in water temperature have also been reported to affect the information encoded by electric cues (Ardanaz et al., 2001). In the present study, we confirmed that while environmental illumination remains constant, water temperature shows daily rhythms that peak close to sunset (Figs 3 and 5). This evidence allows us to postulate that water temperature is the most likely candidate to synchronize the daily rhythm in the natural electric behavior of *G. omarorum*. Given the strong influence temperature has on EOD rate, it is worth noticing that the nocturnal increase of EOD-BR occurs while environmental temperature decreases (Fig. 4), i.e. with an inverted correlation than the expected by the unspecific positive effects of temperature on EOD-BR (Ardanaz et al., 2001). As a consequence, EOD-BR peak does not coincide with the daily peak in temperature (Fig. 5), occurring during the decaying phase of the temperature cycle. In other words, there are at least two different pathways by which temperature exerts its effects on EOD-BR: (1) the non-specific and positive metabolic effect (Ardanaz et al., 2001) and (2) a zeitgeber effect, by which the slight daily variation of water temperature might be used as an environmental cue for the onset of arousal.

Though the role of daily changes in temperature have been postulated as putative circadian entrainers since pioneer studies (Pittendrigh, 1960), there are only very few species in which environmental temperature has been confirmed as a relevant zeitgeber. For example, it has been reported that temperature cycles not only synchronize the circadian clock of *Drosophila* in constant illumination conditions (Busza et al., 2007), but also act as the main circadian zeitgeber in certain misalignments of light and temperature cycles (Harper et al., 2016). This sensitivity to temperature cycles seems to be quite rare in nature. The results presented in this study suggest, for the first time in anamniote vertebrates, the role of water temperature cycle as a circadian zeitgeber that needs to be further explored and confirmed in free-running laboratory conditions.

The daily rhythm in electric behavior is not a consequence of movement, as evidenced by recordings made of motionless fish (Fig. 2). Even though motionless fish have a wider distribution of acrophase values, it is remarkable that the mean population acrophase is similar to the one observed in freely moving fish (recorded in the lagoon under natural vegetation, Fig. 5). This constancy in mean population acrophase is surprising considering that motionless fish were recorded in tanks in which photoperiodic information is available, whereas fish recorded in their natural habitat are in constant darkness conditions. Contrary to our speculation, the availability of photic information did not increase the synchronization among motionless fish which are, in fact, desynchronized in comparison to fish in their natural habitat. In this sense, it might be important to consider the effect of social context in circadian synchronization. Fish recorded in the lagoon are only restricted by a plastic mesh and can perceive other fish electric discharges, while motionless fish are isolated in plastic tanks and are, hence, isolated from conspecifics discharges. Social synchronization of circadian rhythms is currently an area of great interest and debate in chronobiology, one in which electric fish also offer an interesting model.

### Concluding remarks

The nocturnality of electric arousal in *G. omarorum*: (1) is independent of locomotion; (2) persists in constant darkness in the wild; (3) is most likely synchronized by daily variations of both water temperature daily variations and by social cues. *G. omarorum* thus emerges as an exciting wild model system for the study of the circadian rhythms that arise in the complexity of the real world, allowing us to evaluate the integration of environmental physical and social cues in the modulation of the circadian system.

## MATERIALS AND METHODS

Adult *G. omarorum* (Richer-de-Forges et al., 2009) (*n*=17) were used in laboratory (*n*=6) and natural settings, including the recording of six individuals in natural conditions and five individuals isolated in shelters (semi-natural). All specimens were collected in Laguna del Sauce, Depto. de Maldonado (34° 48′ S, 55° 18′ W). Fish were identified using a ‘fish detector’; an electronic audio amplifier connected to a pair of electrodes as described elsewhere (Silva et al., 2003).

The experiments were conducted during the non-breeding season at the peri-equinox period, under a natural light-dark cycle of 12:12. Periodic light and temperature measures were taken each 30 min, inside the water under the natural vegetation and outside the water (HOBO-MicroDAQ: UA-002-08). Measurements range: temperature, −20° to 70°C (−4° to 158°F); light, 0 to 320,000 lux (0 to 30,000 lumens/ft^2^).

All research procedures complied with ASAP/ABS Guidelines for the Use of Animals in Research and were approved by the Institutional Ethical Committee (Comisión de Éticaen el Uso de Animales, Instituto Clemente Estable, MEC, 008/11).

### Exploratory behavior recordings in laboratory settings

With the aim of demonstrating the nocturnality in this species we recorded the presence of exploratory behavior throughout the day. Fish used in these experiments were transported from the collecting site, housed in individual compartments within 500 l outdoor housing tanks and fed *Tubifex tubifex ad libitum*. Recordings were carried out in a naturalistic tank (500 l) containing an ordered array of 12 small shelters (25 cm in length and 3.3 cm in diameter, plastic tubes) each equipped with a pair of electrodes that allowed the recording of the EOD only when animals were sheltered. Animals (*n*=6) were placed individually in the tank and shelters were checked for fish presence once per hour over 24 h. If the fish remained undetected after checking all the shelters it was assumed to be swimming around the tank displaying exploratory behavior.

### EOD-BR recordings in the natural habitat

EOD-BR was recorded from fish (*n*=6) placed in plastic nets with electrodes under the natural vegetation in their natural habitat (Laguna del Sauce) during 72 h. 30 s recordings were made once an hour. Fish in this condition are almost always detectable although they are able to move around and perceive the electric signals of conspecifics.

### EOD-BR recordings in isolated semi-natural settings

Fish (*n*=5) were placed in 50 l individual plastic tanks containing a shelter equipped with a pair of electrodes (as described above). Given the size of the shelters, when recordings show stable EOD amplitudes, we can reliably assume fish remain still while sheltered. EOD-BR was recorded for 30 s per hour over 24 h, if fish were sheltered and hence still. Fish in this condition are isolated from the influence of conspecifics. Light and temperature were monitored as described earlier.

### Data processing and statistical analysis

The EOD was recorded through electrodes placed in the water, digitalized using standard computer soundcards and recorded with a custom developed Matlab (MathWorks, Inc.) program which detects the moment of EOD occurrence.

EOD-BR was calculated as the inverse of the inter EOD intervals in the recordings and expressed in terms of the median±MAD values. As fish differ in their EOD-BR, an index (the BRIn) was calculated to determine the increase between the hour before sunset (BR_−60_) and sunset (BR_sunset_), regardless of absolute values for each fish. The global BRIn for the whole group was calculated as the median±MAD value of individual indexes:
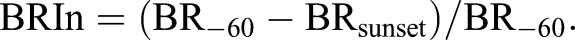


In order to normalize the effect of water temperature on EOD-BR, values were corrected to a constant 20°C temperature by using the Q_10_ value of 1.5 as calculated for electric fish (Dunlap et al., 2000; Silva et al., 2007). Q_10_ is a unitless quantity calculated as the factor by which the rate increases when the temperature (T) is raised by ten degrees Celsius.
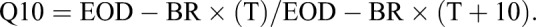


A paired non-parametrical two-tailed Wilcoxon test was used for statistical analysis. Data are shown as median±MAD.

The analysis of the nocturnal increase in EOD-BR was carried out using a cosinor fit (Diez-Noguera, 1999). Cosinor gives a statistical validation for the fitting of a cosine function within a 24 h period to each fish EOD rate data, as well as the acrophase value in lineal hours for each fish that are then transformed to clock hours for simplicity. For animals recorded during a single cycle of 24 h, acrophase was determined by the hour of the maximum EOD-BR value. Population acrophase was calculated and statistically validated using the Rayleigh test with the individual acrophases calculated as described (Refinetti et al., 2007).
